# Serological Evidence of Rift Valley Fever Virus Circulation in Sheep and Goats in Zambézia Province, Mozambique

**DOI:** 10.1371/journal.pntd.0002065

**Published:** 2013-02-28

**Authors:** José Fafetine, Luis Neves, Peter N. Thompson, Janusz T. Paweska, Victor P. M. G. Rutten, J. A. W. Coetzer

**Affiliations:** 1 Veterinary Faculty, Eduardo Mondlane University, Maputo, Mozambique; 2 Biotechnology Centre, Eduardo Mondlane University, Maputo, Mozambique; 3 Department of Production Animal Studies, Faculty of Veterinary Science, University of Pretoria, Onderstepoort, Pretoria, South Africa; 4 Centre for Emerging and Zoonotic Diseases, National Institute for Communicable Diseases of the National Health Laboratory Service, Johannesburg, South Africa; 5 Department of Veterinary Tropical Diseases, Faculty of Veterinary Science, University of Pretoria, Onderstepoort, Pretoria, South Africa; 6 Department of Infectious Diseases and Immunology, Faculty of Veterinary Medicine, Utrecht University, Utrecht, The Netherlands; Centers for Disease Control and Prevention, Kenya

## Abstract

Rift Valley fever (RVF) is endemic in most parts of Africa and has also been reported to occur in the Arabian Peninsula. It is responsible for significant morbidity and mortality, particularly in livestock, but also in humans. During the last two decades several outbreaks of RVF have been reported in countries in Southern Africa. In contrast to other countries, no clinical disease has been reported in Mozambique during this period. In a serological study conducted in 2007 in five districts of Zambézia Province, Mozambique, of a total of 654 small ruminants sampled (277 sheep and 377 goats), 35.8% of sheep sera and 21.2% of goat sera were positive for RVF virus (RVFV) antibodies in a virus neutralization test (VN) and in an IgG enzyme-linked immunosorbent assay (ELISA). In 2010, a cross-sectional survey was conducted in 313 sheep and 449 goats in two districts of the same province. This study revealed an overall seropositivity rate of 9.2% in sheep and 11.6% in goat and an increased likelihood of being seropositive in older animals (OR = 7.3; p<0.001) using an IgG ELISA. 29 out of 240 animals assessed for RVF specific IgM by ELISA were positive, suggesting recent exposure to RVFV. However, a longitudinal study carried out between September 2010 and April 2011 in a cohort of 125 of these animals (74 sheep and 51 goats) failed to demonstrate seroconversion. The results of the study indicate that RVFV circulates sub-clinically in domestic small ruminants in Zambézia Province.

## Author Summary

Rift Valley fever (RVF) is a mosquito-borne disease that results in severe negative impact on human and animal health and the economy. Outbreaks of RVF occur sporadically when heavy rains favour the breeding and emergence of mosquito vectors of the virus. Rift Valley fever has been reported in many African countries and in the Arabian Peninsula; however, in Mozambique there are very few reports of the occurrence of the disease. We conducted a serological study in Zambézia Province, central Mozambique, for the detection of RVFV-specific antibodies in sheep and goats. This study allowed us to gather valuable information on the extent of RVFV infection in animals. Our investigations showed that RVFV-specific antibodies are continuously present in at least a low percentage of sheep and goats, which strongly suggests the circulation of RVFV during inter-epidemic periods without the manifestation of the typical clinical signs. These findings raise the questions of how the virus is maintained and what are the risk factors involved in the maintenance of the virus during the inter-epidemic periods.

## Introduction

Rift Valley fever (RVF) is a disease caused by a RNA virus of the family *Bunyaviridae*, genus *Phlebovirus* [1]. The disease is of considerable economic importance due to high abortion rates, high mortality in young animals, trade restriction and the negative impact on other non-agricultural sectors [2]. The disease is also a serious public health hazard resulting in mild to moderately severe influenza-like illness that may be complicated by ocular lesions, encephalitis or a fatal haemorrhagic state in a low percentage of patients [3].

In the last decade Eastern and Southern African countries have experienced several RVF epidemics responsible for severe losses both in animals and humans [4]–[7]. In 2006/2007 in Kenya, Somalia and Tanzania the disease caused more than 1,000 infections in humans and 323 deaths [4]. In 2008, Madagascar reported an epidemic of RVF that was responsible for at least 476 suspected human cases, 19 deaths and untold high death rates among cattle were also reported [5]. In the same year, an outbreak of the disease, affecting particularly veterinarians and farmers, was reported in South Africa. Of the 53 humans that contracted RVF following exposure to the tissues of sick domestic ruminants, 15% revealed evidence of recent infection and 4% of past exposure to RVFV. Transmission by direct contact as a result of performing necropsies on infected animals was found to be the main risk factor [6]. During an epidemic of RVF in 2010 in South Africa, 192 laboratory-confirmed human cases including 18 deaths were reported [7].

Despite the occurrence of several epidemics of RVF in the past 2 decades in neighbouring countries such as South Africa, no epidemics were reported in Mozambique during this time. There are only a few reports of RVF in Mozambique. In 1960 RVFV antibodies were found in 2.8% of cattle. Nine years later 134 cattle died of RVF in Gaza Province [8]. In 1999 in the Zambézia Province, cases of abortion were reported in a herd of water buffaloes (*Bubalus bubalis*) that were seropositive to RVFV. Surveillance of cattle in the same province in 1996 and 2001 revealed a seroprevalence of 37% and 53%, respectively [9]. A seroprevalence of 2% (28/1163) in humans was found in a study conducted from 1981 to 1983 in 8 of the 10 provinces of Mozambique [10].

The mechanism of virus maintenance during inter-epidemic periods is unclear. The current theory is that RVFV is maintained in aedine mosquito eggs and epidemics occur following an increase in the mosquito population after abnormally heavy rains [11]. It is postulated that the virus is maintained in eggs of *Aedes* mosquitoes that breed in waterlogged depressions called *dambos*. Several mosquito species and biting-flies may act as vectors during an epidemic [3], [12]. Different wildlife species may become infected by RVFV [13], [14]. Evidence of inter-epidemic transmission of RVFV has been shown to occur in African buffalo (*Syncerus caffer*) [15], humans [16], [17] and sheep [18].

Since surveys for RVFV activity are not regularly carried out it is believed that the disease is underreported in Mozambique. There are indications that RVF is endemic in certain parts of the country and that infection occurs in sheep, goats and cattle during the inter-epidemic period [9]. Some districts of the Zambézia Province located in the central part of the country seem to have suitable agro-ecological conditions for the maintenance of the disease. The objective of the present study was to detect circulation of RVFV in sheep and goats in the Zambézia Province in the inter-epidemic period by cross-sectional and longitudinal serological studies.

## Materials and Methods

### Site description

**Figure 1:**
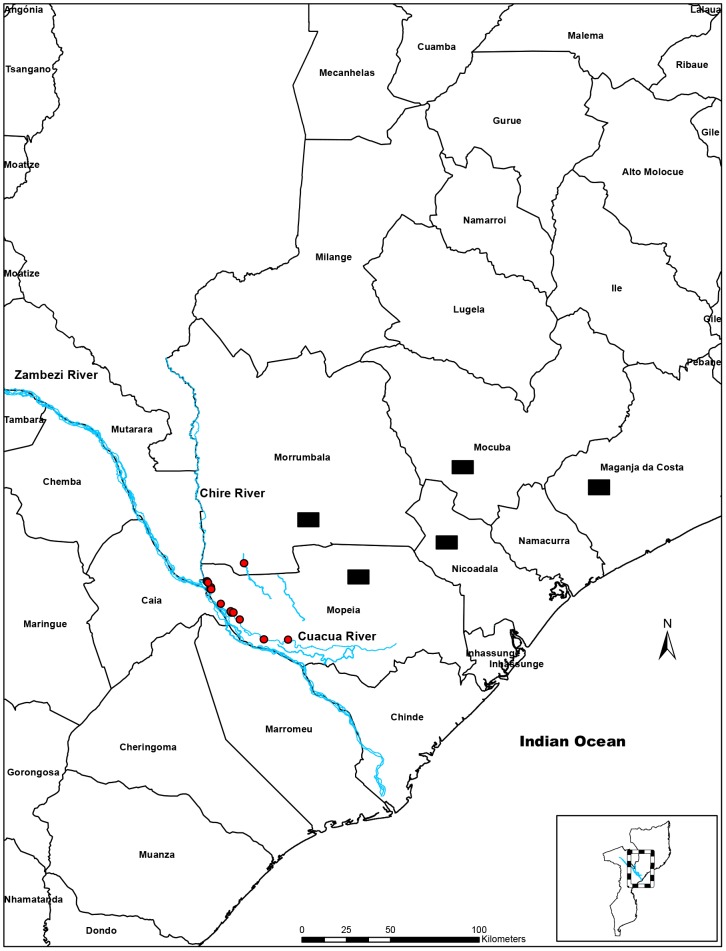
Location of the study areas. Figure 1 shows the map of the Zambézia Province, Mozambique indicating the location of the study areas. In 2007, the collection points for the baseline study were in 5 districts namely Maganja da Costa, Mocuba, Mopeia, Morrumbala and Nicoadala (black rectangle). In 2010, the study was carried out in different locations of the Mopeia (red circle) and in Nicoadala districts. Three perennial rivers that drain the Mopeia district namely Zambeze, Cuacua and Chire Rivers are highlighted in blue.

Zambézia Province is located in the central coastal region of Mozambique (17°0′S; 37°0′E), south of Nampula and north of Sofala Province. It has a total area of 103,127 km^2^, much of it drained by the Zambezi River. The coast consists mainly of mangrove swamps and inland forest. The monthly average minimum temperature in the capital of the province between 1971 and 2000 ranged from 15.3°C to 23.4°C and the average maximum temperature from 26.3°C to 32.4°C. In the same period the average monthly rainfall was 189.4 mm. January, February and March are the months with higher precipitation and July, August, September and October are considered to be the drier months [19]. The province has a total of 32,629 cattle, 1,308 water buffaloes, 194,052 goats and 47,603 sheep, of which the majority are kept in rural areas by subsistence farmers with less than 20 animals each [20]. Five out of 16 administrative districts of the Province, namely, Maganja da Costa, Mocuba, Mopeia, Morrumbala and Nicoadala were chosen for the baseline study in 2007. In September 2010 samples were collected only in Mopeia and Nicoadala districts (Fig. 1).

### Animals and sampling

This study site was selected because no vaccination had been performed in sheep and goats and it was identified by the local Veterinary Services as high risk for RVFV activity based on ecological suitability for the vector, proximity to rivers, presence of *dambos*, historical occurrence of seropositive animals and relatively high concentration of animals [9]. Blood samples were collected from the jugular vein of sheep and goats in plain tubes. Clotted blood samples were separated by centrifugation and the sera stored at −20°C until use. The study consisted of cross-sectional and longitudinal surveys.

### Cross-sectional surveys

In 2007 a cross-sectional survey was conducted as a baseline study in sheep and goats in the five districts previously mentioned. A total of 377 goats and 277 sheep were sampled.

For the estimation of RVF prevalence in Mopeia and Nicoadala districts in 2010, three age groups in sheep and goats were studied, namely 0–6 months (group I), 6–12 months (group II) and more than 12 months old (group III). The sample size was calculated based on an estimated seroprevalence of 50%, maximum allowable error of 10% and confidence level of 95%, giving a required sample size of 97 for each group in both species. Animals were selected using either simple random or systematic random sampling, mainly in small scale farms where the animal owners showed willingness to take part in the survey. At the time of sampling, livestock owners were questioned about any previous occurrence of abortions or other possible signs of RVF in their animals.

### Assessment of inter-epidemic transmission of RVFV

In Mopeia district 125 animals (74 sheep and 51 goats) between 1 and 4 months of age were individually identified using ear-tags. These animals were monitored from September 2010 until April 2011, and bled at 45-day intervals. Additionally, to test for recent infection all the samples that were low positive in the IgG ELISA were further tested for the presence of RVFV-specific IgM.

### Laboratory tests

Virus neutralization test - the virus neutralization (VN) test was conducted in 96-well microplates using Vero cells as described previously [21]. Briefly, duplicates of 25 µl serial two-fold diluted heat-inactivated serum was mixed with an equal volume of 100 TCID_50_ of RVFV (AR 20368 isolate) and incubated at 37°C for 30 min. A total of 50 µl of Vero cells in MEM medium containing 10% foetal bovine sera were then added to each well. The microplates were incubated with 5% CO_2_ and checked daily for the presence of cytopathic effects. The titre was expressed as the reciprocal of the serum dilution that inhibited ≥75% of viral cytopathic effects. A serum sample was considered seropositive when it had a titre of ≥log_10_ 1.0, equivalent to a serum dilution≥1∶10.

IgG indirect ELISA - the IgG indirect ELISA was conducted as described by Fafetine and others [22] and Jansen van Vuren and others [23]. ELISA plates (Maxisorb, Nunc, Denmark) were coated with 100 µl of the recombinant nucleocapsid RVFV diluted 1∶2,000 in carbonate-bicarbonate buffer overnight at 4°C. Plates were washed 3 times with 0,1% Tweeen-20 in phosphate buffer saline (PBS) (washing buffer) and blocked with 200 µl PBS with 10% skim milk. After incubation in a moist chamber for 1 h at 37°C, plates were washed 3 times with washing buffer and 100 µl test, positive and negative control (NICD-SPU) sera diluted 1∶400 in 2% skim milk in PBS were added. Plates were incubated in a moist chamber for 1 h at 37°C, washed as previously described and 100 µl recombinant Protein G HRPO conjugate diluted 1∶15,000 was added (Zymed Lab., Inc). After incubation for 1 h at 37°C, plates were washed 3 times with washing buffer followed by the addition of 100 µl 2,2′-azinodiethylbenzothiazoline sulfonic acid (ABTS, KPL Laboratories, Inc.). Plates were then incubated in dark for 30 min at room temperature, 100 µl of the stop reagent, 1% sodium dodecyl sulphate (SDS) was added to each well and optical densities (OD) were measured at 405 nm. The results were subsequently expressed as percentage of the high positive control serum using the formula [(mean net OD of test serum/mean net OD of high positive control)]×100. The cut-off value previously optimized for the assay (PP values ≥25) was used in this study.

IgM capture ELISA - the IgM capture ELISA was performed following a method previously described by Paweska and others [24]. Briefly, plates were coated overnight at 4°C with 100 µl rabbit anti-sheep IgM (Zymed Laboratories, Inc.) diluted 1∶500 in PBS. After incubation plates were washed three times with the washing buffer (0,1% Tweeen-20 in PBS) and incubated with 10% skim milk in PBS in a moist chamber for 1 h at 37°C. Plates were washed 3 times with the washing buffer and duplicate volumes of 100 µl of test and control sera (NICD-SPU) diluted 1∶400 added in rows A–D; 1–12 respectively to the corresponding wells in the bottom half of the plate (rows E–G: 1–12). After incubation at 37°C for 1 h and washing 6 times with washing buffer, 100 µl of the virus (NICD-SPU) and control antigen (NICD-SPU) diluted 1∶200 in PBS containing 2% skim milk were added to both the rows of the top half of the plate (rows A–D: 1–12) and of the bottom half of the plate (rows E–G: 1–12) respectively. Plates were incubated for 1 h at 37°C, washed 3 times with the washing buffer and mouse anti-RVF serum diluted 1∶3,000 added to each well of the plate. Plates were incubated again for 1 h at 37°C, washed 3 times with the washing buffer and goat anti-mouse IgG conjugated with peroxidase (Zymed laboratories, Inc.) diluted 1∶4,000 added to each well for 1,h at 37°C. Plates were washed 6 times with the washing buffer and the reaction was developed by the addition of ABTS (KPL Laboratories, Inc.). After incubation in a dark at room temperature for 30 min the reaction was stopped by the addition of 100 µl 1% SDS. Optical densities (OD) were determined at 405 nm. The net OD values were first recorded for each serum as the value determined with the RVFV antigen minus the value determined with the control antigen and subsequently converted into percentage of the OD value of a high positive control serum as previously mentioned. Threshold PP values of sheep and goat sera producing PP values ≥7.9 and ≥9.5 respectively, were considered to be positive.

### Statistical analysis

Seroprevalence, with 95% confidence intervals, was calculated for each district and overall, taking into account the different sampling weights in each district. Seroprevalence was compared between districts, and the overall seroprevalence was compared between species, using the chi-square test with second order correction of Rao and Scott to account for the survey design [25]. A multiple logistic regression model was used to estimate the association of age, sex and locality with the outcome (seropositive to RVFV) while controlling for possible confounding. For this purpose, age was categorized into less than 6 months, 7–12 months and more than 12 months, and locality was modelled as a fixed effect. Separate models were used for sheep and for goats. The fit of each logistic regression model was assessed using the Hosmer-Lemeshow goodness-of-fit test. A significance level of 5% was used. Analyses were done using Stata 11 (StataCorp, College Station, TX, USA).

### Ethical approval

The research protocol was approved by the Scientific Board of the Veterinary Faculty of the Eduardo Mondlane University, Maputo, Mozambique. The study permission was obtained from the Mozambican Livestock National Directorate, the Zambézia's Livestock Provincial Directorate, from community leaders and from the farmers. Since most of the animal owners were illiterate, verbal approval for testing their animals was obtained from them after explaining the objectives of the study. In conducting this study, the handling of the animals and the blood sampling were performed by approved staff, namely animal technicians and veterinary surgeons, according to the World Organization for Animal Health (OIE) guidelines for use of animals in research and education.

## Results

### Cross-sectional surveys

Serum samples randomly collected in 2007 in a cross-sectional survey of 377 goats and 277 sheep in five different district of the Zambézia Province were tested with the VN test and indirect IgG ELISA. Antibodies to RVFV were detected in all the districts in both species (except in Mocuba for goats) with the higher seroprevalence values in Nicoadala and Mopeia districts in both species. The adjusted overall prevalence in sheep (35.8%) was higher than in goats (21.2%) (p = 0.0002) (Table 1).

**Table 1:** RVF seroprevalence in 2007, as determined by virus neutralization test and IgG ELISA.

District	Goats			Sheep		
	*n*	Seroprevalence (%)	95% C.I.	*n*	Seroprevalence (%)	95% C.I.
Maganja da Costa	92	39.1^c^	29.7, 49.5	11	54.6^b^	25.6, 80.7
Mocuba	59	0.0^a^	0.0, 4.9	181	13.8^a^	9.5, 19.7
Mopeia	53	50.9^cd^	37.6, 64.1	60	93.3^c^	83.4, 97.5
Morrumbala	131	7.6^b^	4.1, 13.7	–	–	–
Nicoadala	42	61.9^d^	46.3, 75.4	25	80^bc^	59.4, 91.6
**TOTAL**	377	21.2^A^	17.9, 24.9	277	35.8^B^	30.7, 41.1

Table 1 shows RVF seroprevalence in goats and sheep in districts of Zambézia Province, Mozambique.

a,b,c,d Values within a column with no superscripts in common differ significantly (p<0.05).

A,B Seroprevalence differs between goats and sheep (P = 0.0002).

C.I. confidence interval.

In 2010 a total of 449 serum samples from goats and 313 from sheep were collected in seven different localities of the Mopeia and Nicoadala districts. At the time of sampling, no livestock owners could recall ever seeing abortions or other clinical signs of RVF in their animals. The results of the IgG ELISA are shown in Table 2. IgG specific for RVFV was detected in all localities where samples were collected, with seroprevalence ranging from 4.3–50% and the total adjusted seroprevalence in sheep somewhat lower (9.2%) than in goats (11.6%) but not statistically significantly so (p = 0.58). Seroprevalence in sheep was higher in Mopeia (22.8%) than in Nicoadala (4.3%) (p = 0.05). In females apparently higher seroprevalences occurred than in males in both species (Table 3). Seroprevalence was also higher with increasing age.

**Table 2:** RVF seroprevalence in 2010, as determined by IgG ELISA.

Place	Goats			Sheep		
	*n*	Seroprevalence (%)	95% C.I.	*n*	Seroprevalence (%)	95% C.I.
**Mopeia district**	383	13.1	[10.0,16.8]	290	22.8	[18.3,27.9]
Bras	29	6.9^ab^	[1.7,23.9]	–	–	–
Chimuara	140	21.4^b^	[15.4,29.0]	254	21.7^ab^	[16.9,27.2]
Deda	48	12.5^ab^	[5.7,25.2]	8	50.0^b^	[19.9,80.1]
Massancara	6	16.6^ab^	[2.3,63.3]	–	–	–
Nhamirere	53	5.7^a^	[1.8,16.2]	–	–	–
Nuere	53	9.4^ab^	[3.9,20.8]	21	28.6^b^	[13.4,50.9]
Nzanza	54	5.6^a^	[1.8,15.9]	7	14.3^ab^	[1.9,58.4]
**Nicoadala district**	66	10.6^ab^	[5.1,20.8]	23	4.3^a^	[0.6,26.2]
**TOTAL**	449	11.6^A^	[7.7,17.2]	313	9.2^A^	[4.5,17.9]

Table 2 shows RVF seroprevalence in goats and sheep in different localities of Mopeia and Nicoadala districts, Zambézia.

a,b, Values within a column with no superscripts in common differ significantly (P<0.05).

A Seroprevalence does not differ between goats and sheep (P = 0.578).

C.I. confidence interval.

**Table 3:** RVF seroprevalence by sex and age group in 2010.

			Total sampled	No. positive	Seroprevalence (%)
**Goats**	Sex	Female	345	54	15.7
		Male	104	3	2.9
	Age	0–6 months	128	4	3.1
		7–12 months	124	8	6.5
		>12 months	197	45	22.8
**Sheep**	Sex	Female	248	59	23.8
		Male	65	8	12.3
	Age	0–6 months	90	4	4.4
		7–12 months	53	6	11.3
		>12 months	170	57	33.5

Table 3 shows RVF seroprevalence by sex and age group in goats and sheep in Mopeia and Nicoadala districts, Zambézia.

On multiple logistic regression analysis (Table 4) the difference in RVF prevalence between males and females was no longer significant in either species. However, the effect of age was found to be highly significant. In goats, older animals (>12 months) were more likely to be seropositive than animals 0–6 months old (OR = 7.3; p<0.001) and than animals 6–12 months old (OR = 3.3; p = 0.012). Similarly in sheep, older animals (>12 months) were more likely to be seropositive than animals 0–6 months old (OR = 9.5; p<0.001) and than animals 6–12 months old (OR = 5.3; p = 0.001). There was substantial variation in seroprevalence between the localities; compared to Chimuara, goats in Nhamirere had lower odds of being seropositive (OR = 0.23; p = 0.021) and sheep in Deda had higher odds of being seropositive (OR = 5.85; p = 0.035).

**Table 4:** Effect of sex, age and locality on seropositivity to RVFV in 2010.

Species	Variable	Level	Odds ratio (*OR*)	95% confidence interval (*OR*)	*P*-value
Goats	Sex	Female	1*	–	–
		Male	0.34	0.10, 1.20	0.094
	Age	0–6 months	1*	–	–
		7–12 months	2.20	0.60, 8.07	0.234
		>12 months	7.32	2.42, 22.12	<0.001
	Locality	Chimuara	1*	–	–
		Bras	0.30	0.07, 1.40	0.127
		Deda	1.02	0.35, 2.96	0.975
		Massancara	0.60	0.06, 5.65	0.657
		Nhamirere	0.23	0.06, 0.80	0.021
		Nuere	0.44	0.15, 1.25	0.123
		Nzanza	0.30	0.08, 1.09	0.068
		Nicoadala	0.80	0.28, 2.25	0.672
Sheep	Sex	Female	1*	–	–
		Male	0.91	0.37, 2.27	0.853
	Age	0–6 months	1*	–	–
		7–12 months	1.79	0.44, 7.34	0.420
		>12 months	9.50	3.15, 28.67	<0.001
	Locality	Chimuara	1*	–	–
		Deda	5.85	1.13, 30.30	0.035
		Nuere	1.44	0.50, 4.18	0.503
		Nzanza	1.47	0.15, 14.82	0.744
		Nicoadala	0.23	0.03, 1.79	0.158

Table 4 shows the effect of sex, age and locality on seropositivity to RVFV in sheep and goats in Mopeia and Nicoadala districts, Zambézia, in 2010 as determined by multiple logistic regression models.

* Reference level.

To test for recent infection, the IgM ELISA was performed only in the 240 samples that were low positive in the IgG ELISA. Twenty samples from sheep were IgM positive (17 from Chimuara, 2 from Nuere and 1 from Deda) and 9 goat samples were IgM positive (6 from Chimuara, 1 from Nuere, 1 from Nzanza and 1 from Deda).

### Assessment of inter-epidemic transmission of RVFV

The number of animals positive on the IgM and IgG ELISA in the longitudinal study is summarized in Table 5. None of the animals negative at the beginning of the experiment seroconverted during the 7 month study period.

**Table 5:** Number of RVF seropositive animals in the longitudinal study.

	No. positive	
	IgM	IgG
September	5	9
October	1	9
December	1	7*
January	-	5**
April	-	5

* 1 animal slaughtered and 1 animal no longer positive.

** 1 animal slaughtered and 1 animal no longer positive.

## Discussion

In recent years severe outbreaks of RVF have been reported in humans and animals in southern Africa [26]–[29]. Apart from reports of the disease in 1969 [8] and in 1999 [9], as a cause of abortions and deaths in cattle and water buffalo, there are no other records of RVF in Mozambique. However, the Zambézia Province has suitable ecological conditions for the circulation of RVFV. For example, the Mopeia district is drained by three large perennial rivers, namely the Zambeze, Cuacua and Chire, and has extensive wetlands and dambos that provide suitable habitat for mosquito breeding. This study therefore aimed to determine whether RVFV was circulating in the area.

The five districts where the cross-sectional survey was conducted in 2007 were chosen based on reports of the Mozambican Veterinary Services on the possible occurrence of RVFV activity in these districts. The overall detected seroprevalence of RVF in the five districts was 35.8% in sheep and 21.2% in goats. These prevalences are slightly lower than the prevalence of 39% in sheep and 33.5% in goats reported in Comoros in 2009 [30]. A lower prevalence of 24.7% was reported in small ruminants after the 2008 outbreak in Madagascar [28]. Since the diagnostic tests used in the above studies were the same as used in our study, the difference in the prevalence can be attributed to differences in factors related to climate, agro-ecological conditions and/or sampling strategies.

The 2010 survey reported in this study was restricted to Mopeia and Nicoadala because these districts had the highest prevalences in the 2007 study. The overall seroprevalences in both species in the 2010 survey were 9.2% in sheep and 11.6% in goats. The Zambézia Province is characterized by high temperature and humidity, with the rainy season starting in November and ending in April. The rainfall records from the closest meteorological station to the study site show that the average monthly rainfall in 2007 was 150 mm and in 2010 it was 80 mm. The average monthly rainfall from January to April 2007 was 210 mm, compared to 114 mm in 2010. These differences in precipitation may have influenced the different seroprevalence rates obtained since risk factors associated with RVF were found to be water-related e.g. associated with high rainfall and the presence of temporary large surface water bodies [31], [32].

The overall seroprevalence (Table 3) initially suggested a possible influence of sex on the seroprevalence, with a much higher seroprevalence observed in females than in males. However, in the multiple logistic regression model the apparent effect of sex disappeared since it was due to confounding by age. Relatively more of the older animals were females, particularly amongst goats, and adult animals were far more likely to be seropositive than young animals (p<0.001). Thus, the present study demonstrated that the seroprevalence of RVF increased with age of the animals. These results are in agreement with those reported in Mauritania and Senegal [31] and would support a hypothesis of endemic circulation of RVFV in the province, where older animals are more likely to have longer exposure to RVFV than the younger ones. The differences in seroprevalence observed between the localities of Mopeia district in 2010 as well as in different districts in 2007 cannot be answered in this study. Further work to analyze the possible risk factors associated with RVF and RVFV exposure in the different localities are needed.

The IgM ELISA was used to detect recent infections. It has been demonstrated that after experimental infection with RVFV IgM starts to rise after day 3 and at 77 days the levels decrease to that of non-infected animals while IgG was detected from day 5 and remained high until 77 days after infection [22], [24]. In our study, 29 of the 240 animals were IgM ELISA positive, indicating that infection of the animals had probably occurred during the dry season. However, no outbreak of RVF or apparent clinical signs of the disease had been reported during the dry season, or at any time prior to our study, indicating that the cases of RVF infection had been mild or subclinical. This agrees with a study conducted in Madagascar [28] which found evidence of RVFV circulation during the dry season without clinical cases. This finding may indicate either that the virus activity is maintained in the mosquitoes in the dambos that do not dry out completely even during the months with less precipitation allowing a low level of transmission to livestock, or the involvement of other risk factors not related with precipitation in the transmission of RVFV in Zambézia.

To test the hypothesis of endemicity of RVF 125 animals were bled at approximately 45-day intervals in a longitudinal study from September 2010 until April 2011. None of the animals seroconverted. These results show that no active viral transmission occurred in these animals during the study period. Evidence of inter-epidemic transmission has been shown in African buffalo (*Syncerus caffer*) in South Africa where 9 out of 126 seronegative animals seroconverted between 2001 and 2003/4 [15]. The animals in our study were bled for only a 7 month period (middle of the dry season to the end of the rainy season) that was drier than usual, which may have had an effect on our results.

In summary, the presence of antibodies to RVFV in sheep and goats in different districts of Zambézia Province is evidence of inter-epidemic circulation of RVFV with mild or subclinical manifestation. Additional studies including the isolation of the virus from mosquito vector and longitudinal studies in sheep, goats and cattle are necessary to further elucidate the inter-epidemic state of the disease. If one takes into consideration that most rural inhabitants of the sampled districts are subsistence farmers that live in close contact with the animals and may experience the same rate of mosquito exposure, the results presented in this paper suggest that human infection with RVFV may occur regularly in the same areas and may also be an important but overlooked cause of morbidity and mortality.
